# pH-Sensitive Liposomes with Embedded 3-(isobutylamino)cholan-24-oic Acid: What Is the Possible Mechanism of Fast Cargo Release?

**DOI:** 10.3390/membranes13040407

**Published:** 2023-04-04

**Authors:** Anna A. Efimova, Anton S. Popov, Alexey V. Kazantsev, Pavel I. Semenyuk, Irina M. Le-Deygen, Nikolay V. Lukashev, Alexander A. Yaroslavov

**Affiliations:** 1 Department of Chemistry, M.V. Lomonosov Moscow State University, Leninskie Gory 1-3, 119991 Moscow, Russia; 2 Belozersky Institute of Physico-Chemical Biology, Moscow State University, Leninkie Gory 1/40, 119992 Moscow, Russia

**Keywords:** pH sensitivity, liposome, drug delivery, lipid bilayer, cargo release, nanocontainer

## Abstract

pH-sensitive liposomes have great potential for biomedical applications, in particular as nanocontainers for the delivery of biologically active compounds to specific areas of the human body. In this article, we discuss the possible mechanism of fast cargo release from a new type of pH-sensitive liposomes with embedded ampholytic molecular switch (AMS, 3-(isobutylamino)cholan-24-oic acid) with carboxylic anionic groups and isobutylamino cationic ones attached to the opposite ends of the steroid core. AMS-containing liposomes demonstrated the rapid release of the encapsulated substance when altering the pH of an outer solution, but the exact mechanism of the switch action has not yet been accurately determined. Here, we report on the details of fast cargo release based on the data obtained using ATR-FTIR spectroscopy as well as atomistic molecular modeling. The findings of this study are relevant to the potential application of AMS-containing pH-sensitive liposomes for drug delivery.

## 1. Introduction

The application of liposomes as nanocontainers for the encapsulation and delivery of biologically active compounds was suggested decades ago [1,2,3]. Their unique structure allows them to retain both hydrophobic and hydrophilic drugs, they are easy to synthesize and non-toxic, and their composition and size can vary over a wide range, thus showing great potential to be used in biomedicine [4,5,6,7]. Nowadays, much attention is paid to stimulus-sensitive liposomes that are able to release the cargo under the action of an external stimulus [8,9,10]. Among these types of stimuli, pH is of particular interest, since lower pH is typical for tumors, areas of inflammation, etc. [11,12,13]. The application of pH-sensitive liposomes makes it possible to increase the efficiency and therapeutic effect of drug delivery [14]. A great number of pH-sensitive liposomes have been described, but liposomes with various molecular switches in their composition have a significant drawback, i.e., the low rate of release of the encapsulated drug. In most cases, the release process develops within several minutes after acidification [15,16,17,18,19,20]. Recently, there have been extensive attempts to increase the rate of release [17,21]. However, increasing the release rate requires more switches to be included in the membrane, which can lead to an increase in the cytotoxicity of the suggested formulations, as well as in the cost of treatment [20]. Therefore, none of the suggested pH-sensitive systems has evolved into a product. Thereby, it is still a considerable challenge to prepare liposomes that could quickly release the encapsulated substance under small changes in the pH.

**Figure 1:**
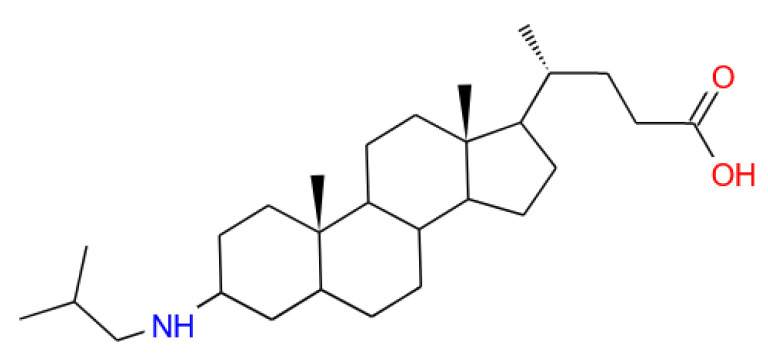
3-(isobutylamino)cholan-24-oic acid (AMS).

Lately, we suggested a new type of pH-sensitive liposomes with embedded ampholytic molecular switch (AMS, 3-(isobutylamino)cholan-24-oic acid) with carboxylic anionic groups and isobutylamino cationic ones attached to the opposite ends of the steroid core (Figure 1).

The inclusion of this AMS into the lipid bilayer led to a significant increase in the cargo release: up to 95% of the internal content of liposomes flowed out in the first minutes after acidification to pH = 6.0 [22]. Apparently, AMS changed its orientation in the liposomal membrane adapting to the acidity/basicity of the outer solution. We assumed that such rotation disrupted the packing of lipids in the membrane and, as a result, led to the rapid release of the encapsulated substance.

The experiments carried out confirm this assumption; however, the exact mechanism of the switch action has not yet been determined. In the present work, in order to determine the cause of the phenomenon described above and to investigate the mechanism of AMS action, we utilized molecular dynamic (MD) simulations in addition to ATR-FTIR spectroscopy experiments. MD simulations have been previously used successfully to study the details of biopolymer interactions with lipid bilayers, which cannot be experimentally obtained: the molecular mechanism and the driving force of the interaction, lipid mobility within membrane leaflets, number of ion pairs, etc. [23,24,25,26,27,28]. This method is often the best tool for explaining the phenomena discovered as a result of the experiments. ATR-FTIR spectroscopy is a highly informative spectroscopy method for the investigation of non-transparent colloidal systems, including lipid vesicles [29,30,31,32]. Recently, its high sensitivity was demonstrated in physicochemical studies of lipid bilayers in complexes with different polycations and in liposomal forms of some drugs. Therefore, in this study, we used ATR FTIR-spectroscopy to track how the state of the lipid bilayer with embedded ampholytic molecular switches changes with the pH of the outer solution. The studies were carried out for liposomes composed of conventional zwitterionic palmitoyloleoylphosphatidylcholine (POPC, Figure S1) that additionally contained AMS 3-(isobutylamino)cholan-24-oic acid.

## 2. Materials and Methods

### 2.1. Chemicals

Electrically neutral 1-palmitoyl-2-oleoyl-glycero-3-phosphocholine (POPC) from Avanti polar lipids) was used as received. 

3-(isobutylamino)cholan-24-oic acid (AMS), which is a derivative of the bile (lithocholic) acid, was synthesized according to Procedure S1, Figures S2–S5 (SI).

The following 10^–3^ M buffer solutions were prepared: tris(hydroxymethyl)aminomethane NH_2_C(CH_2_OH)_3_, fused sodium acetate CH_3_COONa (fresh grade), sodium hydrogen phosphate dodecahydrate Na_2_HPO_4_*12H_2_O, and sodium dihydrogen phosphate NaH_2_PO_4_*2H_2_O. Concentrated HCl and sodium chloride (both chemically pure grade) were used as received. 

Double-distilled water was additionally treated by passing through a Milli-Q system (Millipore, Merck KGaA, Darmstadt, Germany) equipped with ion-exchange adsorption columns for advanced purification from organic impurities, and filters were used for the removal of large particles for the preparation of the solutions. The conductivity of purified water was 0.6 μS/cm.

### 2.2. Synthesis of Liposomes

Small monolamellar liposomes were obtained via sonication. To this end, the required amount of AMS, as well as electroneutral POPC solution (both in a methanol–chloroform mixture) were mixed, and the organic solvent was removed using a vacuum rotary evaporator at 30–35 °C and a rotation speed of 60 rpm. The resulting thin film was dispersed in 1–2 mL of 10^−3^ M of an appropriate buffer solution. Then, the suspension was sonicated with a Cole-Parmer 4710 ultrasonic homogenizer (Vernon Hills, IL, USA) at a frequency of 22 kHz for 600 s (2 × 300 s) at 30 °C. The resulting liposome suspension was separated from titanium dust via centrifugation using a J-11 centrifuge (Beckman, Brea, CA, USA) for 5 min at 11,000 rpm. The molar fraction of AMS was calculated as follows: ν_AMS_ = [AMS]/([AMS] + [POPC]). The value was equal to 0.1.

Liposomes filled with 1 M NaCl were prepared as follows: After removing the solvent, the lipid film was dispersed in a 10^−3^ M buffer with appropriate pH additionally containing 1 M NaCl. The suspension was sonicated and centrifuged as described earlier. The resulting suspension was dialyzed for 1.5 h against 10^−3^ M with appropriate pH renewed every 45 min.

Freshly prepared liposomes with an average hydrodynamic diameter of 30–50 nm (determined via dynamic light scattering) were used in the experiments.

### 2.3. Methods

The average hydrodynamic diameter of the liposomes was estimated using a Brookhaven 90 Plus device (Brookhaven Instruments Company, Holtsville, NY, USA) at a fixed angle of 90 °C. Light intensity fluctuations were recorded using a Brookhaven 90 Plus correlator (Brookhaven Instruments Company, USA). The hydrodynamic radii were calculated using the Stokes equation in the approximation of spherical particles.

The electrophoretic mobility of the liposomes was measured using laser microelectrophoresis using a Brookhaven 90 Plus device (Brookhaven Instruments Company, USA) in a thermostated cell according to the standard method proposed by the manufacturer.

The leakage of salt from the liposomes to the surrounding solution was monitored via the measurement of the suspension conductivity since it is accompanied by an increase in the electrical conductivity of the suspension. The measurements were carried out using a CDM 83 conductometer (Radiometer, Copenhagen, Denmark). Polyethylene cuvettes were used for the measurements. The sample volume was 1 mL. The maximum increase in electrical conductivity Ω_max_ was achieved after the addition of a 10-fold excess of Triton X-100 surfactant, which completely destroyed the liposomes. The obtained value Ω_max_ was taken as 100%, and the relative increase in electrical conductivity was measured as the ratio Ω/Ω_max_, %.

The pH of the solutions was evaluated using a Hanna 210 pH meter (Hanna Instruments, Vöhringen, Germany) equipped with an HI 1131B glass measuring electrode.

### 2.4. Molecular Dynamic (MD) Simulations

A CHARMM36m force field [33,34] was used for molecular dynamic simulations; three forms of AMS molecule (cationic, zwitterionic, and anionic) were parameterized using the CGenFF service [35]. A simulation box 10 × 10 × 12 nm with a bilayer of 304 POPC molecules and 16 embedded AMS molecules (8 molecules in each layer) was prepared using the CHARMM-GUI web service [36]; the AMS molecule was aligned to the z-axis (i.e., perpendicular to the bilayer). Then, for each form of AMS, two systems with a different orientation of the AMS molecule were generated: with the amino group oriented outwards (AMS*_NH2top) and the carboxylic group oriented outwards (AMS*_COOtop) in all AMS molecules (Table 1).

After energy minimization and equilibration, the main 100 ns simulations with the step of 2 fs were performed using the GROMACS 5.1 software [37]. Periodic boundary conditions and the particle mesh Ewald method were used for handling long-range electrostatic interactions. The simulations were conducted using an NPT ensemble. A Nose–Hoover thermostat [38,39] and the Parrinello–Rahman pressure coupling algorithm [40] were used.

### 2.5. ATR-FTIR Spectroscopy Experiments

The spectra were recorded using a Tensor 27 ATR-FTIR spectrometer (Bruker, Bremen, Germany) equipped with an MCT detector cooled with liquid nitrogen and a thermostat (Huber, Offenburg, Germany). The measurements were carried out in a BioATR II thermostated cell (Bruker, Germany) using a single reflection ZnSe element at 22 °C and under the continuous purging of the system with dry air using a compressor (JUN-AIR, Redditch, UK). An aliquot (50 µL) of the corresponding solution was applied to the internal reflection element, the spectrum was recorded three times in the range of 4000 to 950 cm^−1^ with a resolution of 1 cm^−1^; then, 70-fold scanning and averaging were performed. The background was registered in the same way and was automatically subtracted using the program. The spectra were analyzed using the Opus 7.0 software, Bruker.

## 3. Results and Discussion

### 3.1. POPC/AMS Liposomes

AMS was embedded into the membrane of the POPC liposomes according to the procedure described above. The size of the mixed POPC/AMS liposomes, measured via DLS, lay within a 30–50 nm interval, while electrophoretic mobility (EPM), a parameter associated with their surface charge, was equal to −1.61 (µm/s)/(V/cm) for the liposomes prepared in an alkaline environment and +2.98 (µm/s)/(V/cm) for those obtained in an acidic medium. For studying the stability of POPC/AMS liposomes upon dilution, a 2 mg/mL suspension of liposomes was progressively diluted with aqueous buffers (acidic and alkaline) down to 0.2 mg/mL; meanwhile, the size (hydrodynamic diameter) and surface charge (EPM) of the liposomes were measured. It was found that the liposomes were stable at different pH values: Upon dilution with aqueous buffers (acidic and alkaline), the size and EPM of the POPC/AMS liposomes remained virtually unchanged and were equal to the corresponding values for the liposomes in the initial undiluted suspension (see Table 2 and Table 3). This result definitely showed that the liposomes were stable and not destroyed in either alkaline or acidic media and that AMS did not leave the membrane of the POPC/AMS liposomes under dilution.

The POPC/AMS liposomes also demonstrated aggregative stability at physiological salt concentrations. In the buffer with 0.15 M NaCl, the size of the liposomes remained virtually unchanged for at least 12 h after preparation. 

**Figure 2:**
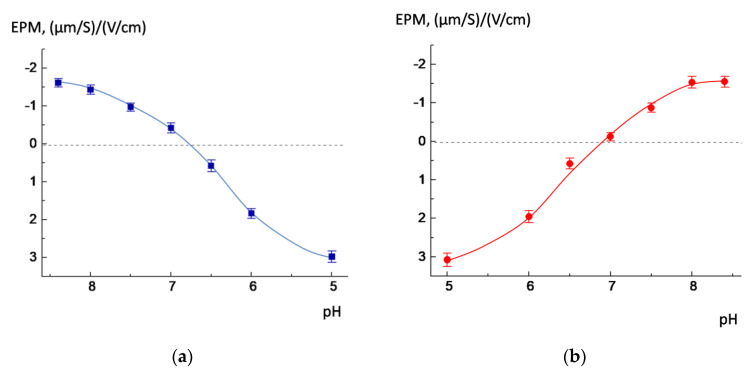
The EPM of POPC/AMS liposomes (α_AMS_ = 0.1) prepared in a pH 8.4 buffer (**a**) and in a pH 5.0 buffer (**b**) and then transferred to buffers with different pH values. Lipid concentration 1 mg/mL. α_AMS_ = 0.1. The dashed line indicates the value of the EPM that is equal to zero.

In the next step, we investigated how the EPM of the liposomes changed upon the acidification or alkalization of the outer solution (Figure 2). The POPC/AMS liposomes were obtained in a buffer with pH 8.5 and then moved to buffers with lower pH values: from 8 down to 5 (Figure 2a). After that, the experiment was repeated in reverse: The liposomes were prepared at pH 5.0 and transferred to buffers with higher pH values (Figure 2b). When the outer solution was progressively acidified, the liposomes lost their negative charge and then became positively charged: The EPM of the liposomes ranged from −1.61 (µm/s)/(V/cm) in an alkaline environment to +2.98 (µm/s)/(V/cm) in an acidic environment (Figure 2a). The mirror situation was observed during alkalization (Figure 2b). The liposomes became neutral at pH between 6.7 and 6.9. A question arises: Do liposomes retain their size after moving from an alkaline to an acidic solution and vice versa? The DLS measurements revealed that the liposomal size did not practically change in the pH interval from 8.4 down to 5 and from 5 to 8.4 and remained within the range of 30−50 nm, which was evidence of undisrupted liposomes in both alkaline and acidic media.

### 3.2. Release of the Cargo by POPC/AMS Liposomes under the Changes in the pH

**Figure 3:**
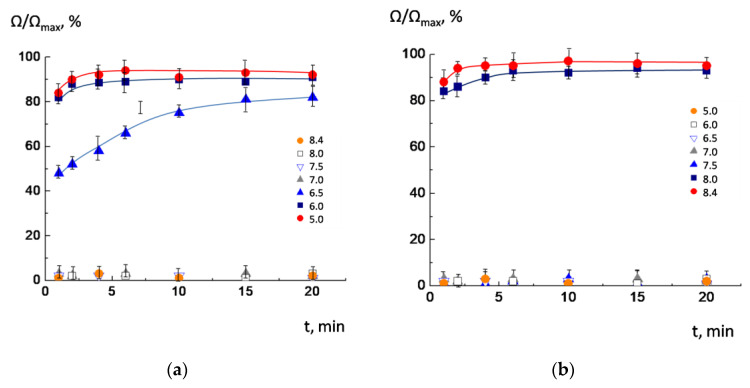
Time-dependent changes in the relative electrical conductivity of NaCl-loaded POPC/AMS liposomes at different pH values. Liposomes were prepared at pH = 8.4 (**a**) and at pH = 5.0 (**b**) and then transferred to buffers with different pH. Lipid concentration 1 mg/mL. α_AMS_ = 0.1.

Recently, we found that POPC/AMS liposomes effectively release cargo after acidification: Up to 85–95% of the internal content of liposomes flowed out in the first minutes after decreasing pH to 6.0 [22], while there was no release after decreasing pH down to 8.0, 7.5, and 7.0 (Figure 3a). The release induced by slight acidification of the outer medium is of special interest from a biomedical point of view: typically, the measured pH values for pathological areas lie in the range of 5.9–6.9 [11,12,13].

We decided to investigate whether the release also takes place in a “mirror” situation: NaCl-loaded POPC/AMS liposomes were prepared in an acidic buffer with pH = 5.0 and transferred to buffer solutions with higher pH values. The results of the experiment are shown in Figure 3b. As inferred from the figure, the release of salt began at pH = 8.0 and 8.4 (curves 6 and 7), when the surface charge in the liposomes changed from positive to negative due to the dissociation of carboxyl groups. However, the liposomes retained their integrity at pH 5.0, 6.0, 6.5, 7.0, and 7.5 (curves 1–5). It should be noted that the rate of salt outflow was also effective: 90–95% of salt was released from the liposomes within the first minutes.

Comparing the results shown in Figure 2 and Figure 3, one could infer that the salt release began when the liposomes’ EPM approached a charge opposite to the initial. We attributed this phenomenon to the correlation between the new charge, caused by the rotation of the switch molecule in the liposomal membrane, which led to the formation of defects and subsequent leakage of sodium chloride from the internal cavity of the liposomes. It was also shown that the incorporation of anionic lipid cardiolipin into the membrane did not affect the functioning of the switch: The liposomes effectively released 90% of the cargo in the first 3–5 min upon acidification (Figure S6). In the next stage of the investigation, we attempted to reach a more accurate understanding of the exact mechanism of the switch action via atomistic molecular modeling as well as by means of ATR-FTIR spectroscopy.

### 3.3. Molecular Modeling of the Switch

Aiming to obtain molecular insight into the switching mechanism, we carried out atomistic molecular modeling of the behavior of three forms of AMS: the anionic form (AMS^−^) realized in an alkaline medium, the zwitterionic form (AMS^0^) realized at neutral pH, and the cationic form (AMS^+^) realized in an acidic environment due to the protonation of amino groups. All three forms were embedded into a lipid bilayer in different (opposite) orientations, resulting in two sets of simulations: the first one (AMS*_NH2top) with the amino groups looking outwards and the second one (AMS*_COOtop) with the amino groups looking inwards (Table 1).

**Figure 4:**
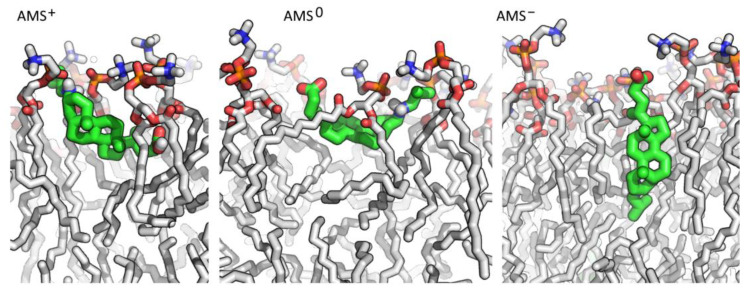
Typical poses of the cationic, zwitterionic, and anionic forms of AMS in the lipid membrane. AMS and lipid molecules are shown in stick representation and colored according to the atom types: green and gray for carbons in AMS and lipids, respectively; polar hydrogen atoms of AMS are colored in white; other hydrogen atoms are hidden.

**Figure 5:**
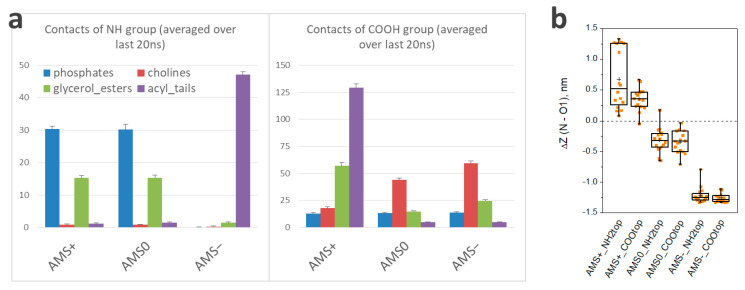
(**a**) Number of contacts between amino group or carboxylic group of AMS and different groups of lipids. Values are averaged for each form of AMS; standard error is shown; (**b**) vertical component of the distance between the amino group and carboxylic group; each point represents a single AMS molecule.

The simulations resulted in different orientations of the different forms of AMS, and the final orientation was independent of the initial orientation of AMS molecules. Typical poses are presented in Figure 4. Indeed, the optimal orientation of the anionic form, AMS^−^, was vertical with the negatively charged carboxyl group -COO^−^ located in the outer part of the bilayer and interacting with the choline groups of lipids. The other part of the molecule, including the neutral amino group, was immersed into the hydrophobic part of the bilayer, efficiently forming hydrophobic interactions (Figure 5a). Few rare cases of side-by-side interaction between two AMS^−^ molecules were observed (no more than 2 pairs in each system with 16 AMS molecules), but the stability and probability of these contacts did not indicate any significance. By contrast, the zwitterionic form was oriented horizontally in the outer (polar) part of the bilayer. Charged amino and carboxylic groups interacted with phosphate (and probably also with ester groups) and choline groups of lipids, respectively. Finally, the cationic form, AMS^+^ was oriented so that the amino group would be located in the outer region of the protonated (neutral) carboxylic group. The protonated amino group was located close to the bilayer surface and strongly interacted with lipid phosphates, whereas the neutral carboxylic group was located close to the inner (hydrophobic) part of the bilayer and interacted with ester groups (Figure 5a). A few molecules of AMS^+^ stayed in a vertical orientation throughout the simulation and formed two interlayer pairs that interacted via neutral carboxylic groups: the total length of two AMS molecules was similar to the bilayer thickness.

In summary, the optimal orientation of AMS molecules in the membrane strongly depended on the protonation state, suggesting the capability of the rotation caused by pH alteration. The difference between the z-coordinates of amino groups and carboxylic groups of AMS unambiguously indicates how pronounced the changes can be (Figure 5b). Thus, the z-component of the distance between the two mentioned groups in the anionic form was 1.3 nm, which almost coincided with the actual distance between these groups, indicating a vertical orientation of the molecule. By contrast, the zwitterionic form was oriented almost horizontally, while the cationic form was oriented with an opposite tilt: When protonated, the carboxylic group of AMS moved from the outer part of the bilayer inwards closer to the lipid ester groups. Notably, being embedded in a “wrong” vertical orientation, the AMS molecule rapidly (in a few nanoseconds) rotated (Figure S7), and the final orientation did not depend on the initial one, thus indicating that the simulations reached an equilibrium state. The fact that in AMS^+^_NH2top simulation, few molecules did not reach the optimal orientation might reflect a slow kinetics of the switching or a probable opportunity to form intra-layer contacts between two molecules of AMS, which consequently might affect the membrane integrity. However, orientations of the cationic and anionic forms of AMS enabled a significant level of hydrophobic interaction with non-polar tails of the lipids (in contrast to the zwitterionic form), though this seemed to be different in different forms, and consequently, switching from AMS^+^ to AMS^−^ and vice versa required strong rotation of the molecule and alteration in hydrophobic interactions.

### 3.4. ATR-FTIR Spectroscopy

To disclose changes in the microenvironment of lipids caused by AMS rotation, we used ATR-FTIR spectroscopy, a powerful method providing valuable data on the bilayer state. On the typical ATR-FTIR spectrum of liposomes, a few bands are the most informative.

The most pronounced bands correspond to the asymmetric and symmetric valence oscillations of methylene groups ν as (CH_2_) at 2917–2925 cm^−1^ and ν as (CH_2_) at 2850–2852 cm^−1^ are sensitive to changes in lipid packing in bilayer [41]. The bands of carbonyl groups ν(CO) at 1720–1750 cm^−1^ and phosphate groups ν as (PO_2_^−^) at 1220–1260 cm^−1^ are usually multi-component: High-frequency components correspond to lower-hydration groups, and low-frequency groups correspond to high hydration [31,42,43]. Both of these bands are sensitive to the interaction of the membrane with polar ligands.

**Figure 6:**
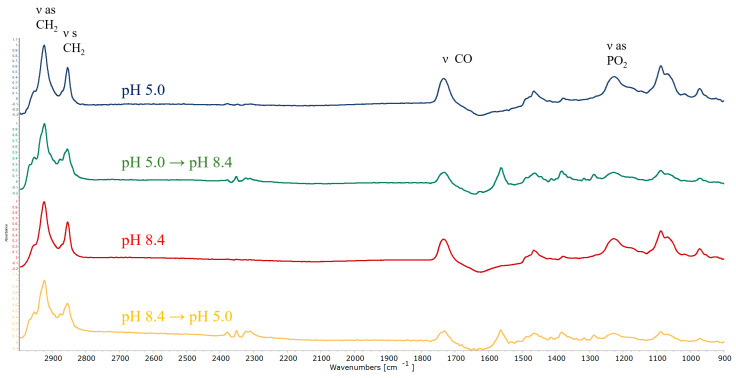
Normalized ATR-FTIR spectra of POPC/AMS liposomes in various media. Total lipid concentration 5 mg/mL, α_AMS_ = 0.1, 22 °C.

Figure 6 shows a general view of the spectra of the liposomes in acidic and alkaline media (blue and red lines). With the exception of small differences in the region of the absorption bands of the polar groups (carbonyl and phosphate), apparently caused by the difference in their protonation, the spectra do not differ significantly. The spectral pattern corresponds to liquid crystal liposomes at room temperature.

**Figure 7:**
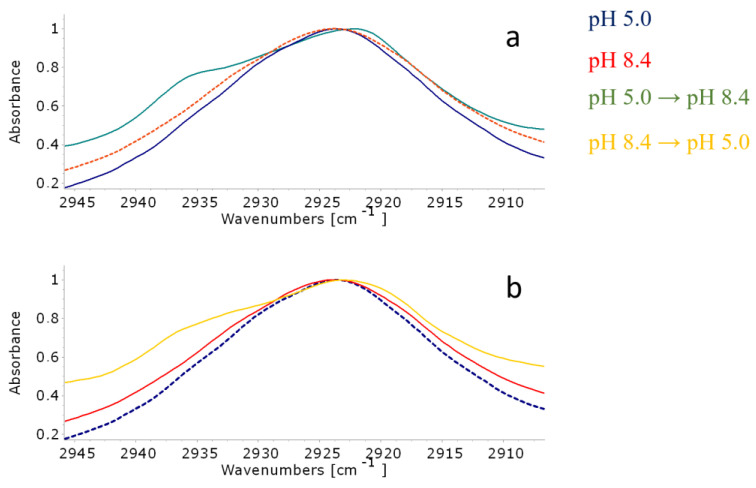
Normalized ATR-FTIR spectra of POPC/AMS liposomes (absorption region of asymmetric stretching vibrations of methylene groups) under various conditions: (**a**) liposomes at pH 5.0 (blue), at pH 8.4 (red dashed line), and converted from pH 5.0 to 8.4 (green); (**b**) liposomes at pH 5.0 (blue dashed line), at pH 8.4 (red line), and converted from pH 8.4 to pH 5.0 (yellow). Total lipid concentration 5 mg/mL, α_AMS_ = 0.1, 22 °C.

According to molecular modeling, when changing from an acidic to an alkaline media, AMS molecules rotated and could probably perturb the bilayer. This process is reflected in the spectra in the splitting of the absorption band of asymmetric stretching vibrations of methylene groups: A shoulder appears at 2935 cm^−1^ (Figure 7a), which is characteristic of the most mobile hydrophobic chains [44]. Previously, a similar splitting effect was observed during, for example, the mechanical loosening of the membrane under the action of vibrations of magnetic nanorods in an ultralow-frequency non-heating magnetic field [32].

It should be noted that the spectrum of the liposomes subjected to medium change did not correspond to that of the liposomes in an alkaline medium (green line VS red dashed line, Figure 7a). Indeed, this spectrum reflected the non-equilibrium state of the bilayer, in which, apparently, there were regions with increased mobility of hydrophobic chains. Potentially, these sites serve as pores for releasing the contents of the liposomes.

We observed a similar but less intense effect when the liposomes were transferred from an alkaline to an acidic medium (Figure 7b). Again, the spectrum of such liposomes did not correspond to edge equilibrium states. A shoulder was observed at 2935 cm^−1^, though less pronounced than for the opposite transition but still statistically significant. Such spectral changes indicate that the switch, when turned, actually caused changes in the molecular organization of the bilayer, which is in good agreement with the data of other experiments and the results of molecular modeling.

When it comes to gentler changes in media (from pH 5.0 to neutral and back, and from pH 8.4 to neutral and back), no significant changes in the lipid bilayer package were observed: The bands of ν as (CH_2_) remained mostly without changes (Figures S8–S11). This result is in good agreement with data from cargo release: indeed, bilayer perturbation in the mild condition was not enough for significant liposome leakage.

For a more accurate description of the process of membrane loosening, we used the procedure of deconvolution of the absorption band of asymmetric stretching vibrations of methylene groups (Figures S12–S14). Indeed, the transitions from an acidic to an alkaline environment and vice versa led to a statistically more significant loosening, reflected in an increase in the integral fraction of the 2933 cm^−1^ component. Additionally, vice versa, when changing into or out of a neutral medium, the effect was most often not enough for significant loosening. Thus, the data from deconvolution confirm the results of experiments and mathematical modeling.

## 4. Conclusions

Here, we investigated the possible mechanism of fast cargo release of a new type of pH-sensitive liposome with embedded ampholytic molecular switch (AMS, 3-(isobutylamino)cholan-24-oic acid). Molecular modeling and ATR-FTIR spectroscopy elucidated the details of the switch action. AMS, being embedded into the membrane of POPC liposomes, changed its orientation in the bilayer, thus adapting to the acidity/basicity of the outer solution. The transfer of the cationic form to anionic as well as of the anionic form to the cationic one induced a pronounced rearrangement of the AMS molecules in the lipid membrane. Such transitions caused significant changes in the hydrophobic part of the bilayer that involves lipid tails. This was accompanied by the disordering of the bilayer and the formation of temporal defects in the membrane followed by a subsequent release of the encapsulated substance. At the same time, the transfer of the cationic or anionic form to the zwitterionic one did not induce a pronounced disordering of the lipid membrane. Apparently, the last transition is not associated with serious rearrangements in the hydrophobic part of the bilayer. These findings are promising for the potential biomedical application of POPC/AMS pH-sensitive liposomes.

## Figures and Tables

**Table 1:** The composition of the simulated systems.

	Amino Group	Carboxylic Group	Counterions
AMS^+^_NH2top	>NH_2_^+^	–COOH	4x Na^+^, 20x Cl^–^
AMS^+^_COOtop	>NH_2_^+^	–COOH	4x Na^+^, 20x Cl^–^
AMS^0^_NH2top	>NH_2_^+^	–COO^–^	20x Na^+^, 20x Cl^–^
AMS^0^_COOtop	>NH_2_^+^	–COO^–^	20x Na^+^, 20x Cl^–^
AMS^−^_NH2top	>NH	–COO^–^	20x Na^+^, 4x Cl^–^
AMS^−^_COOtop	>NH	–COO^–^	20x Na^+^, 4x Cl^–^

**Table 2:** EPM and particle size for POPC/AMS liposomes of different concentrations (α = 0.1) obtained in a buffer with pH 8.4.

	Total Lipid Concentration, mg/mL					
	2	1	0.8	0.6	0.4	0.2
**EPM, (μm/s)/(V/cm)**	−1.61 ± 0.11	−1.61 ± 0.11	−1.58 ± 0.08	−1.59 ± 0.07	−1.62 ± 0.08	−1.63 ± 0.06
**Hydrodynamic diameter** **, nm**	45 ± 5	42 ± 4	39 ± 2	44 ± 2	39 ± 4	43 ± 5

**Table 3:** EPM and particle size for POPC/AMS liposomes of different concentrations (α = 0.1) obtained in a buffer with pH 5.0.

	Total Lipid Concentration, mg/mL					
	2	1	0.8	0.6	0.4	0.2
**EPM, (μm/s)/(V/cm)**	2.97 ± 0.07	2.98 ± 0.05	2.98 ± 0.06	2.98 ± 0.07	2.99 ± 0.08	3.00 ± 0.07
**Hydrodynamic diameter** **, nm**	36 ± 6	39 ± 5	40 ± 3	35 ± 3	47 ± 3	36 ± 5

## Data Availability

The data are available from the corresponding author upon request.

## Supplementary Materials

The following supporting information can be downloaded at: https://www.mdpi.com/article/10.3390/membranes13040407/s1, Figure S1: Structure of 1-palmitoyl-2-oleoyl-glycero-3-phosphocholine (POPC); Procedure S1: AMS synthesis; Figure S2: Methyl 3β-(isobutylamino)-5β-cholan-24-oate (^1^H NMR, CDCl_3_); Figure S3: Methyl 3β-(isobutylamino)-5β-cholan-24-oate (^13^C NMR, CDCl_3_); Figure S4: 3β-(Isobutylamino)-5β-cholan-24-oic acid (^1^H NMR, CD_3_OD) (AMS); Figure S5: 3β-(Isobutylamino)-5β-cholan-24-oic acid (^13^C NMR, CD_3_OD) (AMS); Figure S6: Time-dependent changes in the relative electrical conductivity of NaCl-loaded POPC/CL/AMS liposomes at different pH values. Liposomes were prepared at pH = 8.4 (a) and at pH = 5.0 (b) and then transferred to buffers with different pH. Lipid concentration 1 mg/mL. αAMS = 0.1; Figure S7: Time dependences of the position of the amino group and carboxylic group of AMS averaged among AMS molecules in each lipid leaflet; Figure S8: Normalized ATR-FTIR spectra of POPC/AMS liposomes in various media. Total lipid concentration 5 mg/mL, α_AMS_ = 0.1, 22 °C; Figure S9: Normalized ATR-FTIR spectra of POPC/AMS liposomes (absorption region of asymmetric stretching vibrations of methylene groups) under various conditions. **a.** Liposomes at pH 5.0 (turquoise), at pH 7.2 (yellow), and converted from pH 7.2 to 5.0 (red) and from pH 5.0 to 7.2 (blue). Total lipid concentration 5 mg/mL, α_AMS_ = 0.1, 22 °C; Figure S10: Normalized ATR-FTIR spectra of POPC/AMS liposomes in various media. Total lipid concentration 5 mg/mL, α_AMS_ = 0.1, 22 °C; Figure S11: Normalized ATR-FTIR spectra of POPC/AMS liposomes (absorption region of asymmetric stretching vibrations of methylene groups) under various conditions. Liposomes at pH 7.2 (blue), at pH 8.4 (green), and converted from pH 7.2 to 8.4 (yellow) and from pH 8.4 to 7.2 (red). Total lipid concentration 5 mg/mL, αAMS = 0.1, 22 °C; Figure S12: ν as (CH_2_) band deconvolution for the samples of POPC/AMS liposomes with initial pH 5.0: **a.** Distribution of integral shares of components 2923 cm^−1^ (blue) and 2933 cm^−1^ (ginger). Deconvolution of the spectrum at **b.** pH 5.0, **c**. transferred to pH 7.2, and **d.** transferred to pH 8.4. Total lipid concentration 5 mg/mL, α_AMS_ = 0.1, 22 °C. Curve-fitting parameters: Gauss shape, *p*-value < 0.005; Figure S13: ν as (CH_2_) band deconvolution for the samples of POPC/AMS liposomes with initial pH 7.2: **a.** Distribution of integral shares of components 2923 cm^−1^ (blue) and 2933 cm^−1^ (ginger). Deconvolution of the spectrum at **b.** pH 7.2, **c**. transferred to pH 5.0, and **d.** transferred to pH 8.4. Total lipid concentration 5 mg/mL, α_AMS_ = 0.1, 22 °C. Curve-fitting parameters: Gauss shape, *p*-value < 0.005; Figure S14: ν as (CH_2_) band deconvolution for the samples of POPC/AMS liposomes with initial pH 8.4: **a.** Distribution of integral shares of components 2923 cm^−1^ (blue) and 2933 cm^−1^ (ginger). Deconvolution of the spectrum at **b.** pH 8.4, **c**. transferred to pH 5.0, and **d.** transferred to the pH 7.2. Total lipid concentration 5 mg/mL, α_AMS_ = 0.1, 22 °C. Curve-fitting parameters: Gauss shape, *p*-value < 0.005.
