# Supplementary material for: pH-Sensitive Liposomes with Embedded 3-(isobutylamino)cholan-24-oic Acid: What Is the Possible Mechanism of Fast Cargo Release?
Source: Membranes (Basel). 2023 Apr 4;13(4):407. doi: 10.3390/membranes13040407 (PMC10141028; doi:10.3390/membranes13040407)
Supplement: Supplementary file 1 [file membranes-13-00407-s001.zip › Supplementary-2303989-revised.doc]

*Supplementary materials for article*

**pH-Sensitive liposomes with embedded 3-(isobutylamino)cholan-24-oic acid: what is the possible mechanism of fast cargo release?**

**Anna A. Efimova ^1,^*, Anton S. Popov ^1^, Alexey V. Kazantsev^1^, Pavel I. Semenyuk^2^, Irina M. Le-Deygen^1^, Nikolay V. Lukashev^1^ and Alexander A. Yaroslavov^1^**

^1^	Department of Chemistry, M.V. Lomonosov Moscow State University, Leninskie Gory 1-3, 119991 Moscow, Russia

^2^	Belozerskii Institute of Physico-Chemical Biology, Moscow State University, Leninkie Gory 1/40, 119992 Moscow, Russia

*****	Correspondence: ephimova@belozersky.msu.ru; Tel.: +74959393116

POPC

CL

**Figure S1.** Structures of 1-palmitoyl-2-oleoyl-glycero-3-phosphocholine (POPC, 1) and cardiolipin (CL, 2)

**Procedure S1.** AMS synthesis.

**Methyl 3β-(isobutylamino)-5β-cholan-24-oate** was synthesized according:

To a mixture of 97 mg (0.25 mmol) methyl 3β-amino-5β-cholan-24-oate and 25 μl (19.8 mg, 0.275 mmol) isobutyraldehyde in 6 ml of dry 1,2-dichloroethane 80 mg (0.375 mmol) of sodium triacetoxyborohydride was added at ambient temperature and obtained solution was stirred for 24 h at ambient temperature. The reaction mixture was diluted with CH_2_Cl_2_ (30 ml), washed twice with sat. aqueous solution of Na_2_CO_3_. Organic layer was separated, dried with Na_2_SO_4_ and solvents evaporated. The product was purified by column chromatography (CH_2_Cl_2_/MeOH 10:1). Yield – 91%, white powder. ^1^H NMR (400 MHz, CDCl_3_), δ: 3.66 (s, 3H, COOCH_3_), 2.95 (bs, 1H, 3α-CH), 2.43 (d, *J* = 6.7 Hz, 2H, NCH_2_), 2.39-2.17 (m, 2H, 23-CH_2_), 1.99-1.75 (m, 6H), 1.67-1.02 (m, 21H), 0.96 (s, 3H, 19-CH_3_), 0.93 (d, *J* = 6.7 Hz, 6H, (CH_3_)_2_CH), 0.91 (d, *J* = 6.5 Hz, 3H, 21-CH_3_), 0.64 (s, 3H, 18-CH_3_).^13^C NMR (100 MHz, CDCl_3_): 174.7, 56.6, 55.9, 54.8, 52.9, 51.4, 42.7, 40.2, 40.0, 36.6, 35.6, 35.3, 35.1, 31.0, 30.3, 30.3, 28.2, 27.7, 26.9, 26.2, 24.1, 24.0, 23.8, 21.0, 20.9, 20.8, 18.2, 12.0.

**3β-(Isobutylamino)-5β-cholan-24-oic acid (AMS)** was synthesized according:

In a vial with a screw cap 59 mg (0,132 mmol) of methyl 3β-(isobutylamino)-5β-cholan-24-oate was placed and 5 ml of 1,4-dioxane with 5% aq. KOH (1:1) added. Obtained mixture was stirred for 3 h at 80°C, evaporated to the half of volume and acidified by HCl to pH 5-6. Product was extracted by EtOAc (3x30 ml), organic layer separated and dried with Na_2_SO_4_ and solvents were evaporated. Yield – 97%, white powder. ^1^H NMR (400 MHz, CD_3_OD), δ:3.46 (bs, 1H, 3α-CH), 2.86 (d, *J* = 6.9 Hz, 2H, NCH_2_), 2.38-1.08 (m, 29H), 1.05 (d, *J* = 6.5 Hz, 6H, (CH_3_)_2_CH), 1.04 (s, 3H, 19-CH_3_), 0.94 (d, *J* = 6.3 Hz, 3H, 21-CH_3_), 0.70 (s, 3H, 18-CH_3_). ^13^C NMR (100 MHz, CD_3_OD): 176.2, 57.7, 57.4, 57.0, 54.2, 43.9, 41.4, 41.3, 37.5, 36.9, 36.7, 36.0, 32.3, 32.1, 30.7, 29.2, 28.4, 27.4, 27.0, 26.7, 25.2, 23.6, 22.5, 22.1, 20.5, 18.8, 12.0. MALDI-TOF: 432.3966 [M+H]^+^. Calculated for C_28_H_50_NO_2_: 432.3836.

**Figure S2.** Methyl 3β-(isobutylamino)-5β-cholan-24-oate (^1^H NMR, CDCl_3_)

**Figure S3.** Methyl 3β-(isobutylamino)-5β-cholan-24-oate (^13^C NMR, CDCl_3_)

**Figure S4.** 3β-(Isobutylamino)-5β-cholan-24-oic acid (^1^H NMR, CD_3_OD) (AMS)

**Figure S5.** 3β-(Isobutylamino)-5β-cholan-24-oic acid (^13^C NMR, CD_3_OD) (AMS)

NMR spectra were recorded with a Bruker Avance 400 (1H 400 MHz, 13C 100.6 MHz) spectrometer at ambient temperature. Chemical shifts are presented in ppm (δ scale) and referenced to tetramethylsilane (δ=0 ppm) in the 1H NMR spectra and to the solvent signal in the 13C NMR spectra. MALDI-TOF spectra were recorded with a Bruker Daltonics UltraFlex instrument in a dithranol matrix using PEG 400 or PEG 600 as the internal standard.

| (**a**) | (**b**) |
| --- | --- |

**Figure S6.** Time-dependent changes of the relative electrical conductivity of NaCl-loaded POPC/CL/AMS liposomes at different pH values. Liposomes were prepared at pH=8.4 (**a**) and at pH=5.0 (**b**) and then transferred into buffer with different pH. Lipid concentration 1mg/ml. α_AMS_ = 0.1.

**Figure S7.** Time dependences of the position of amino group and carboxylic group of AMS averaged among AMS molecules in each lipid leaflet.

**Figure S8.** Normalized ATR-FTIR spectra of POPC/AMS liposomes in various media. Total lipid concentration 5 mg\ml, α_AMS_ = 0.1, 22◦C.

**Figure S9**. Normalized ATR-FTIR spectra of POPC/AMS liposomes (absorption region of asymmetric stretching vibrations of methylene groups) under various conditions. **a.** Liposomes at pH 5.0 (turquoise), at pH 7.2 (yellow), and converted from pH 7.2 to 5.0 (red) and from pH 5.0 to 7.2 (blue). Total lipid concentration 5 mg\ml, α_AMS_ = 0.1, 22◦C.

**Figure S10.** Normalized ATR-FTIR spectra of POPC/AMS liposomes in various media. Total lipid concentration 5 mg\ml, α_AMS_ = 0.1, 22◦C.

**Figure S11**. Normalized ATR-FTIR spectra of POPC/AMS liposomes (absorption region of asymmetric stretching vibrations of methylene groups) under various conditions. **a.** Liposomes at pH 7.2 (blue), at pH 8.4 (green), and converted from pH 7.2 to 8.4 (yellow) and from pH 8.4 to 7.2 (red). Total lipid concentration 5 mg\ml, α_AMS_ = 0.1, 22◦C.

**Figure S12**. ν as (CH_2_) band deconvolution for the samples of POPC/AMS liposomes with initial pH 5,0. **a.** Distribution of integral shares of components 2923 cm^-1^ (blue) and 2933 cm^-1^ (ginger). Further:  Deconvolution of spectrum at **b.** pH 5,0. **c**. Transferred to the pH 7,2. **d.** Transferred to the pH 8,4. Total lipid concentration 5 mg\ml, α_AMS_ = 0.1, 22◦C. Curve-fitting parameters: Gauss shape, p-value < 0,005.

**Figure S13**. ν as (CH_2_) band deconvolution for the samples of POPC/AMS liposomes with initial pH 7,2. **a.** Distribution of integral shares of components 2923 cm^-1^ (blue) and 2933 cm^-1^ (ginger). Further:  Deconvolution of spectrum at **b.** pH 7,2. **c**. Transferred to the pH 5,0. **d.** Transferred to the pH 8,4. Total lipid concentration 5 mg\ml, α_AMS_ = 0.1, 22◦C. Curve-fitting parameters: Gauss shape, p-value < 0,005.

**Figure S14**. ν as (CH_2_) band deconvolution for the samples of POPC/AMS liposomes with initial pH 8,4. **a.** Distribution of integral shares of components 2923 cm^-1^ (blue) and 2933 cm^-1^ (ginger). Further:  Deconvolution of spectrum at **b.** pH 8,4. **c**. Transferred to the pH 5,0. **d.** Transferred to the pH 7,2. Total lipid concentration 5 mg\ml, α_AMS_ = 0.1, 22◦C. Curve-fitting parameters: Gauss shape, p-value < 0,005.
